# Mental health state and its determinants in German university students across the COVID-19 pandemic: findings from three repeated cross-sectional surveys between 2019 and 2021

**DOI:** 10.3389/fpubh.2023.1163541

**Published:** 2023-05-09

**Authors:** Angeliki Tsiouris, Antonia M. Werner, Ana N. Tibubos, Lina M. Mülder, Jennifer L. Reichel, Sebastian Heller, Markus Schäfer, Lisa Schwab, Thomas Rigotti, Birgit Stark, Pavel Dietz, Manfred E. Beutel

**Affiliations:** ^1^Department of Psychosomatic Medicine and Psychotherapy, University Medical Center of the Johannes Gutenberg University Mainz, Mainz, Germany; ^2^Department of Psychology, Goethe-University Frankfurt/Main, Frankfurt, Germany; ^3^Nursing Science, Diagnostics in Healthcare and E-Health, University of Trier, Trier, Germany; ^4^Department of Work, Organizational, and Business Psychology, Johannes Gutenberg University Mainz, Mainz, Germany; ^5^Institute of Occupational, Social and Environmental Medicine, University Medical Center of the Johannes Gutenberg University Mainz, Mainz, Germany; ^6^Department of Communication, Johannes Gutenberg University Mainz, Mainz, Germany; ^7^Institute for Sport Science, Sports Medicine, Rehabilitation and Disease Prevention, Johannes Gutenberg University Mainz, Mainz, Germany; ^8^Leibniz Institute for Resilience Research, Mainz, Germany

**Keywords:** university students, depressive and anxiety symptoms, loneliness, suicidal ideation, mental health, COVID-19 pandemic

## Abstract

**Background:**

Students were at an increased risk for elevated mental symptoms during the first year of the COVID-19 pandemic compared to pre-pandemic levels. As universities remained closed much longer than anticipated, the mental burden was expected to persist through the second year of the pandemic. The current study aimed to investigate the prevalence of mental distress from 2019 through 2021 and identify risk factors for elevated mental burden, focusing on gender.

**Methods:**

We analyzed three cross-sectional online surveys among students at the University of Mainz, conducted in 2019 (*n* = 4,351), 2020 (*n* = 3,066), and 2021 (*n* = 1,438). Changes in the prevalence of depressive symptoms, anxiety, suicidal ideation, and loneliness were calculated using Pearson's chi-square tests and analyses of variance. Multiple linear regressions yielded associated risk factors.

**Results:**

The proportion of students with clinically relevant depressive symptoms was significantly higher during the pandemic (38.9% in 2020, and 40.7% in 2021), compared to pre-pandemic (29.0% in 2019). Similarly, more students reported suicidal ideation and generalized anxiety during the pandemic with a peak in the second pandemic year (2021). The level of loneliness was significantly higher in 2020, compared to 2019, and remained at a high level in 2021 (*p* < 0.001, $\eta_{\text{p}}^{2}$ = 0.142). Female and diverse/open gender, being single, living alone, and being a first-year student were identified as risk factors associated with mental burden during the pandemic.

**Discussion:**

Mental burdens remained elevated among students through the second year of the pandemic and were associated with socio-demographic risk factors and pandemic-related concerns. Future research should monitor recovery and evaluate the need for psychosocial support.

## Introduction

With the beginning of the COVID-19 pandemic, research focused on the mental adaptation of different populations to these novel and uncertain circumstances. Young adults (1–4) and students (5–7) were one of the most adversely affected groups during the pandemic, with increased proportions of depressive symptoms (8–15), suicidal ideation (15, 16), anxiety (8, 10, 12–15, 17), and loneliness (9, 12) compared to pre-pandemic.

In a longitudinal analysis (*n* = 443) of data from our students' health initiative “Healthy Campus Mainz,” we documented a small but significant increase in depressive symptoms and a medium increase in loneliness from pre-pandemic (2019) to during the pandemic (2020) (9). A further cross-sectional analysis revealed an increasing prevalence of Internet addictions from 2019 to 2020, with depressive symptoms, anxiety, and loneliness as significantly associated variables and gender-related differences (18). Several systematic reviews and meta-analyses including international studies corroborated high levels of mental burden in different student populations after the onset of the pandemic. Meta-analyses indicated elevated levels of depressive symptoms with pooled prevalence ranging from 26 to 39% (19–23). Similarly, 29–41% of students indicated high anxiety levels (20, 21, 24, 25).

Until now, data on students' mental health during the further course of the pandemic are scarce as many studies compared pre-pandemic data to pandemic data collected at one measuring time, mostly in 2020. A German survey among 5,642 university students reported results of two cross-sectional samples collected during the pandemic in 2020/2021 and revealed a significant increase in the severity of depressive symptoms and alcohol and drug consumption, as well as higher numbers of students reporting loneliness and suicidal ideation in 2021 (26). This finding underlines that mental health symptoms in the first year of the pandemic did not only persist but even aggravated in the second year.

Li et al. (27) summarized prior research on factors associated with mental burden in students during the pandemic in a systematic review and meta-analysis. In addition to psychological and health behavior variables, women showed higher levels of depressive symptoms and anxiety, underlining the well-known relevance of gender as a risk factor. Analyses of diverse gender students' experience of mental burden are often neglected, although preliminary evidence has indicated that sexual/gender minority students were at high risk of increased anxiety and depressive symptoms during the pandemic (8, 26).

### Research objectives

The current study reports data from three large cross-sectional samples and compares pre-pandemic mental health in 2019 (*n* = 4,351) with mental health during the COVID-19 pandemic in 2020 (*n* = 3,066) and 2021 (*n* = 1,438). As the pandemic closure of university campuses persisted much longer than anticipated covering four full semesters, this study was undertaken to comprehensively assess the mental health burden (depressive symptoms, anxiety, suicidal ideation, and loneliness) from 2019 to 2021 and identify risk factors in the first 2 years of the pandemic, with a focus on the role of gender, study experience, and living situation. Female gender is known to be a risk factor for mental symptoms in the general population (4, 28) as well as in students (7, 9, 12). Similarly, diverse gender was shown to be related to more mental health issues during the pandemic (29, 30). Regarding study experience (being a first-year student or already an advanced student), it can be expected that due to closed universities with only online lectures and no campus life, new students might not get the chance to develop a social network and, therefore, might be more prone not only to loneliness, an acknowledged risk factor to mental health (31, 32), which increased during the pandemic (33), but also to depressive symptoms, and anxiety, compared to students who can already lean on several persons in their network (fellow students, friends, and lecturers and professors). Similarly, students living alone (neither with parents/family members or friends) should be more at risk for experiencing mental distress during the pandemic, as well as students who were not in a romantic partnership.

## Materials and methods

### Data collection and study design

The study was part of the interdisciplinary student health initiative “Healthy Campus Mainz” at Johannes Gutenberg University (JGU) Mainz. This project assessed data on health conditions, health behaviors, and associated determinants in three large surveys performed in the summer terms 2019 to 2021. Approval was obtained by the ethical committees of the Medical Association of Rhineland-Palatinate for the first study (No. 2019-14336), and the Institute of Psychology of the JGU for the second and third study (No. 2020-JGU-psychEK-S008 and 2021-JGU-psychEK-S017). Details on the study methodology (including study design, recruitment, and participants) are reported elsewhere (34).

The study population included students at JGU Mainz, with ~31,500 students in 10 faculties (e.g., medicine, philosophy and philology, biology, law, and business) and two schools (Mainz School of Music and Mainz Academy of Fine Arts). Participants were excluded if they had missing values on the main outcome scales. Using a central mailing list, all students were invited to the respective surveys, and reminder e-mails were sent two times. Furthermore, we promoted the surveys in lectures and with promotional material distributed at the university campus (leaflets, posters, postcards, etc.). While face-to-face study promotion was still possible in 2019 (including promotion on campus), online study promotion was predominant in 2020 and 2021 due to the pandemic. Participation was voluntary, and participants provided informed consent via click-to-agree before starting the online survey (www.unipark.com) (34).

The questionnaires in 2020 and 2021 differed from those presented in 2019 as these surveys included COVID-19-specific items. In the current study, we refer to the 2019, 2020, and 2021 surveys as pre-pandemic, first, and second pandemic year, as it was not until 2020 that the COVID-19 pandemic had an impact on everyday life in Germany. The first pandemic wave in Germany lasted from March to May 2020, followed by a summer plateau (May 2020 to August/September 2020) and a second and third pandemic waves from October 2020 to June 21. The 2021 summer plateau lasted from June 2021 to July/August 2021 (35).

Data were collected in June and July 2019, June and July 2020 (after the first German lockdown that lasted from March 2020 to May 2020), and June through August 2021 (after the second German lockdown that lasted from December 2020 to January 2021). The partial lockdowns brought many restrictions on public life (e.g., closure of schools, universities, restaurants, cinemas, and retail) in Germany. However, in contrast to other European countries (e.g., Italy), it was always possible to meet at least one person.

As both pandemic surveys were conducted during the summer plateaus with a lower incidence of COVID-19 cases, these survey periods were marked by comparably light protective measures. While wearing a face mask was mandatory on public transportation and indoors, it was possible to engage in most recreational activities, such as visiting restaurants and bars, traveling, and meeting up with larger groups during the summer months in 2020 and 2021.

All three surveys queried an individual participant code in order to identify students who repeatedly took part in the study for longitudinal analyses, as reported, for instance, in Werner (9). As the number of participants with matching questionnaires from 2019 through 2021 was small (*n* = 95), we decided to only conduct cross-sectional analyses in the current study.

### Measures

All data were assessed via self-report questionnaires. Demographic and study-related variables included age, first-year student status, being in a partnership, and living situation. Categories indicating gender were “male,” “female,” and “diverse” in the questionnaires 2019 and 2020. In the 2021 survey, we added a fourth response option, “open,” based on the choice given in the German civil register. In order to compare gender throughout the three surveys, responses in the 2021 survey on “diverse” and “open” were summarized into one response category, “diverse/open.” Furthermore, dummy-coded variables for female gender (0 = “male and diverse/open gender,” 1 = “female gender”) and diverse/open gender (0 = “male and female gender,” 1 = “diverse/open gender”) were computed to conduct regression analyses. We categorized students as first-year students if they indicated their current university study as the first study and reported no more than two university semesters, resulting in the dichotomous variable 0 = “not a first-year student” and 1 = “first-year student.” Partnership status was assessed providing three response options (1 = “single,” 2 = “in a partnership with a common household,” 3 = “in a partnership with a separate household”). The dichotomous variable indicating being in a partnership was computed with 0 = “not in a partnership” and 1 = “in a partnership” (summarizing response options “2” and “3”). Living with others was assessed with five response options (1 = “living with parents/family,” 2 = “living in a student dormitory,” 3 = “living alone in an apartment,” 4 = “living together with a partner/spouse,” and 5 = “living in a shared apartment”). We dichotomized the variable assessing living situation into “living with others” (0 = “no” and 1 = “yes”), with “no” representing the answer 3=“living alone in an apartment,” and “yes” summarizing the remaining response options.

Depressive symptoms were assessed with the Patient Health Questionnaire 9 (PHQ-9) (36). This nine-item questionnaire measures symptoms of major depression (e.g., loss of interest and suicidal ideation) over the last 2 weeks on a scale ranging from 0 = “not at all,” 1 = “several days,” 2 = “on more than half of the days,” to 3 = “nearly every day.” A sum score ≥10 is interpreted as at least moderate depressive symptoms. The psychometric properties of the PHQ-9 are very good (37). Suicidal ideation is indicated by one item of the PHQ-9 (“Over the last 2 weeks, how often have you been bothered by thoughts that you would be better off dead or of hurting yourself in some way”). We dichotomized suicidal ideation as 0 = “no suicidal ideation” and 1 = “any level of suicidal ideation” (summarizing responses “on single days,” “on more than half of the days,” and “almost every day”). The two-item GAD-2 (Generalized Anxiety Disorder-2) (38) measured feelings of worry and nervousness with a response scale analogous to the PHQ-9. A sum score ≥3 represents generalized anxiety. The psychometric properties of the GAD-2 are acceptable (38). The German version of the three-item questionnaire UCLA loneliness scale (39) was used to assess loneliness. Participants were asked to indicate how often they (1) feel a lack of companionship, (2) feel isolated from others, and (3) feel left out on a 5-point Likert scale ranging from 0 = “never” to 4 = “very often.” The UCLA loneliness scale has good psychometric properties (39–41).

Pandemic-related experiences were assessed with self-developed and evaluated items. Participants indicated whether they had concerns about isolation/quarantine, their own health, the health of a close person infected with COVID-19, supply shortages, and the economic impact of the pandemic on a 7-point Likert scale ranging from 1 = “no fear at all,” via 4 = “neutral,” to 7 = “very strong fear.” Participants also indicated perceived stress due to not pursuing hobbies, not seeing family, not seeing friends, canceled/postponed medical appointments, and if they needed psychological support on a scale ranging from 1 = “not at all” to 5 = “very.” A principal component analysis (PCA) conducted in an earlier analysis by Werner et al. (9) yielded a two-factor structure. The component of *social stress* was comprised of the stress of not meeting friends, not seeing important family members, not pursuing hobbies, fear of isolation/quarantine, and concern for the economic consequences of the pandemic. The component *health issues* summarized concern for one's health, concern for an infected person, concern for canceled/postponed medical appointments, fear of supply shortages, and the need for psychological support. The overall scores of the scales of *social stress* and *health issues* were calculated using z-standardized values.

### Statistical analyses

Descriptive statistics, including means and proportions, were calculated to describe the study population. Cross-sectional comparisons of proportions across 2019 to 2021 were conducted using chi-square tests for variables with a categorical level. Continuous variables were analyzed with analyses of variance (ANOVA), with the year (2019, 2020, 2021) serving as the independent variable and the variable of interest as the dependent variable. *Post-hoc* analyses were conducted to identify significant differences. Effect size partial eta square was interpreted as ${\eta_{\text{p}}}^{2}$ ≥ 0.01 small, ${\eta_{\text{p}}}^{2}$ ≥ 0.06 medium, and ${\eta_{\text{p}}}^{2}$ ≥ 0.14 large effect. Multiple regression analyses were run to determine associated factors for mental health parameters (depressive symptoms, anxiety, and loneliness) in 2020 and 2021. We included female and diverse/open gender (dummy-coded), relationship status, study progress, living situation, and pandemic-related social stress and health issues as predicting variables. All statistical analyses were performed with IBM SPSS, version 27.

## Results

After data cleaning, we analyzed data of *n* = 4,351 completed questionnaires in 2019, *n* = 3,066 in 2020, and *n* = 1,438 in 2021. Response rates based on the total number of university students in the semester decreased over time, with 14.0%, 10.0%, and 4.7% for the surveys in 2019, 2020, and 2021, respectively. Table 1 shows the sample characteristics of the three surveys. Across measurements, female students were predominant (70.5%−74.2%), and the mean age ranged from 23.4 to 23.8. Over 50% were in a relationship in 2019 and 2020, and 50% in the second pandemic year (2021).

**Table 1:** Sample characteristics in the three surveys.

	**2019 *N* = 4,351**	**2020 *N* = 3,066**	**2021 *N* = 1,438**	**Statistical tests**	**Significant group differences**
***n*** **(%)/*****M*** **(SD)**	***n*** **(%)/*****M*** **(SD)**	***n*** **(%)/*****M*** **(SD)**		
Gender					
Female	3,065 (70.5%)	2,225 (72.6%)	1,065 (74.2%)	χ^2^ = 122.897, *p* ≤ 0.001^a^	
Male	1,246 (28.6%)	821 (26.8%)	338 (23.5%)		
Diverse	39 (0.9%)	20 (0.7%)	12 (0.8%)		
Open	n.a.	n.a.	21 (1.5%)		
Age (*M*/SD)	23.76 (4.35)	23.38 (4.34)	23.60 (4.57)	*F*_(2,8452)_ = 6.658, *p* = 0.001, ${\eta_{\text{p}}}^{2}$ = 0.002	2019–2020 (*p* = 0.001)
In a partnership	2,350 (54.1%)	1,520 (53.0%)	717 (50.0%)	χ^2^ = 7.016, *p* ≤ 0.05	
Living with others	3,352 (84.5%)	2,546 (88.7%)	1,129 (86.6%)	χ^2^ = 24.323, *p* ≤ 0.001	
Being a first year student	723 (17.1%)	664 (22.1%)	286 (20.8%)	χ^2^ = 29.936, *p* = < 0.001	
Clinically relevant depressive symptoms	1,249 (29.0%)	1,117 (38.9%)	536 (40.7%)	χ^2^ = 104.560, *p* ≤ 0.001	
Any level of suicidal ideation	691 (16.0%)	470 (16.3%)	282 (21.4%)	χ^2^ = 26.778, *p* ≤ 0.001	
Generalized anxiety	1,272 (29.7%)	983 (34.2%)	472 (35.9%)	χ^2^ = 26.003, *p* ≤ 0.001	
Loneliness (M/SD)	3.86 (2.69)	6.27 (2.88)	5.87 (3.07)	*F*_(2,8452)_ = 702.06, *p* < 0.001, ${\eta_{\text{p}}}^{2}$ = 0.142	2019–2021 (*p* < 0.001)
					2020–2021 (*p* < 0.001)
					2019–2020 (*p* < 0.001)

Values may not sum up to the total n of the respective year due to missing values. The effect size was estimated by partial eta square (${\eta_{\text{p}}}^{2}$); with ${\eta_{\text{p}}}^{2}$ ≥ 0.01 indicating a small, ${\eta_{\text{p}}}^{2}$ ≥ 0.06 a medium, and $\eta_{\text{p}}^{2}$ ≥ 0.14 a large effect.

a Chi-square analysis conducted with the gender variable that summarizes diverse and open gender in 2021 into one category (diverse and open), resulting in three categories: “male,” “female,” and “diverse/open gender”.

### Depressive symptoms, anxiety, loneliness, and suicidal ideation across 2019, 2020, and 2021

The proportion of students with clinically relevant depressive symptoms was significantly higher during the pandemic (38.9% in 2020 and 40.7% in 2021), compared to pre-pandemic (29.0% in 2019). Similarly, more students reported generalized anxiety in 2021 (35.9%) compared to 2019 (29.7%, *p* ≤ 0.001). Loneliness was higher in 2020 than in 2019 and remained elevated in 2021 (*p* ≤ 0.001). With an effect size of ${\eta_{\text{p}}}^{2}$ = 0.142, differences in the levels of loneliness over the three surveys represent a large effect. The proportion of students reporting any level of suicidal ideation was at a similar level in 2019 (16.0%) and 2020 (16.3%), but significantly higher in 2021 (21.4%, *p* ≤ 0.001).

ANOVAs conducted with the continuous PHQ-9 variable (depressive symptoms) as the dependent variable and year as the independent variable yielded a significant effect [*F*_(2,8494)_ = 94.493, *p* = 0.001, ${\eta_{\text{p}}}^{2}$ = 0.022]. *Post-hoc* analyses revealed significant differences between 2019 vs. 2020 [*M* (SD) =7.41 (5.16) vs. *M* (SD) = 8.91 (5.63)] and 2019 vs. 2021 [*M* (SD) = 7.41 (5.16) vs. *M* (SD) = 9.22 (5.64)]. The same pattern emerged for the continuous GAD-2 variable (generalized anxiety) as the dependent variable and year as the independent variable [*F*_(2,8475)_ = 12.937, *p* = 0.001, ${\eta_{\text{p}}}^{2}$ = 0.003]. *Post-hoc* analyses identified significant differences between 2019 vs. 2020 [*M* (SD) = 1.97 (1.63) vs. *M* (SD) = 2.11 (1.77)] and 2019 vs. 2021 [*M* (SD) = 1.97 (1.63) vs. *M* (SD) = 2.22 (1.78)].

**Figure 1:**
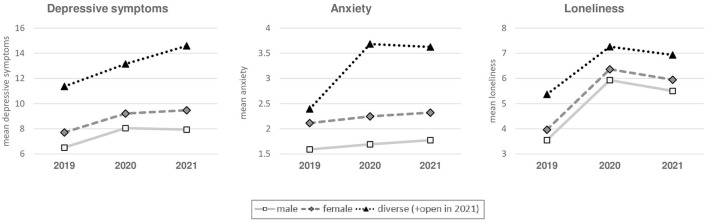
Anxiety, loneliness, and depressive symptoms based on gender and year. Gender assessed in the 2021 survey consisted of four response options: male, female, diverse, and open. For these analyses, response options diverse and open were summarized into one category diverse/open.

ANOVAs analyzing mental burden by time and gender revealed significant main effects of time and gender, illustrated in Figure 1. The main effect of gender on depressive symptoms [*F*_(2,8454)_ = 65.249, *p* = 0.001, ${\eta_{\text{p}}}^{2}$ = 0.015], anxiety [*F*_(2,8454)_ = 83.674, *p* = 0.001, ${\eta_{\text{p}}}^{2}$ = 0.019], and loneliness [*F*_(8,8454)_ = 22.358, *p* = 0.001, ${\eta_{\text{p}}}^{2}$ = 0.005] indicates different levels of mental burden within gender across all surveys. Students with diverse/open gender had the highest values in all mental distress parameters. In addition, female students had significantly higher values in depressive symptoms, anxiety, and loneliness than male students.

**Figure 2:**
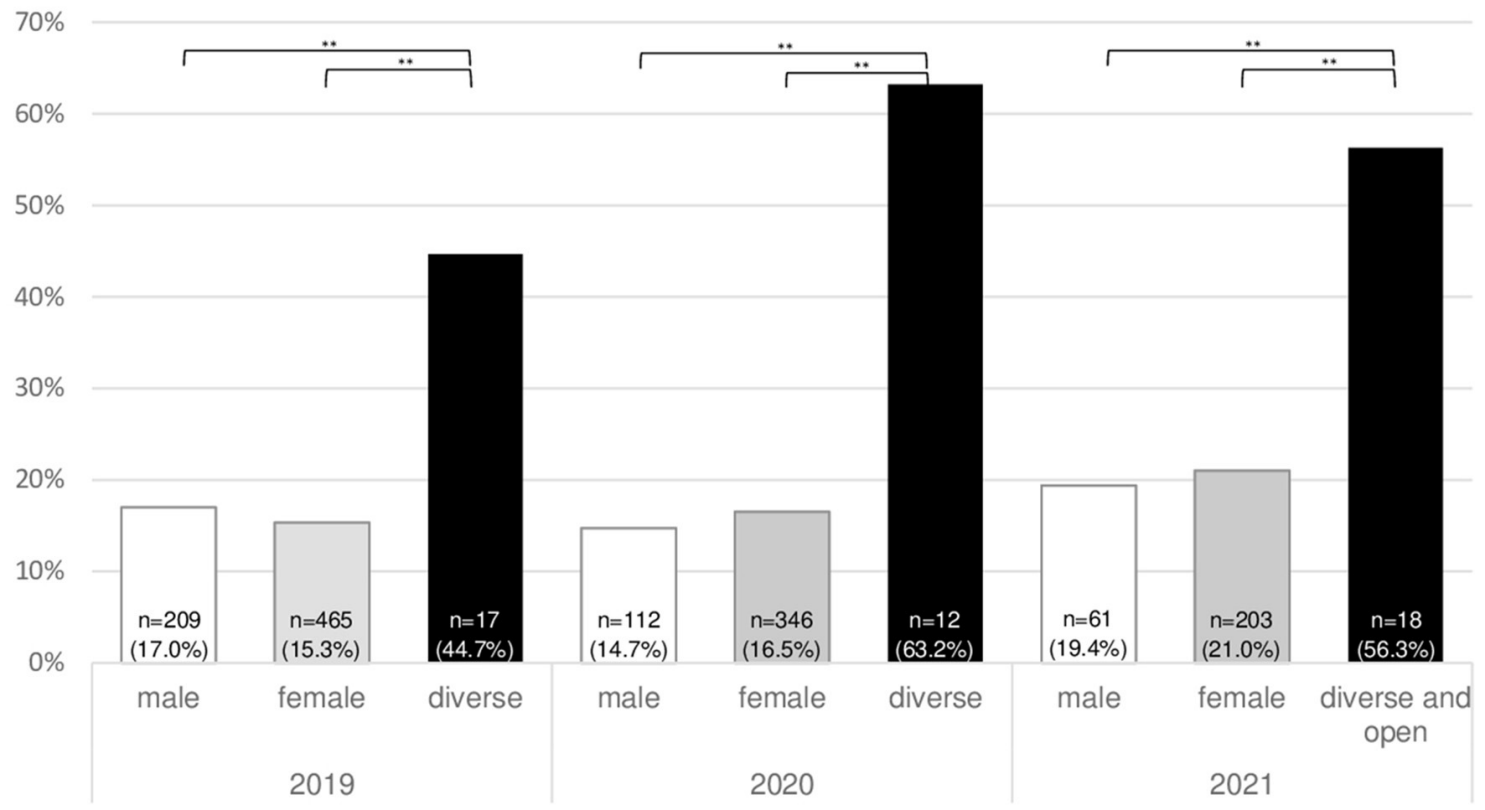
Proportions of students within each gender category reporting any degree of suicidal ideation. **Statistical significance at the *p* < 0.001 level.

In terms of suicidality by year and gender, we observe the highest proportions of students reporting any level of suicidal ideation among diverse/open-gender students, with a peak of 63.2% in 2020 (Figure 2). Suicidal ideation among male and female students ranged from 15.3% in 2019 to 21.0% in 2021.

### Factors associated with depressive symptoms, anxiety, and loneliness in 2020 and 2021

Associated factors for depressive symptoms, anxiety, and loneliness are reported in Table 2. Female and diverse/open gender represented risk factors for depressive symptoms in 2020, while in 2021, only diverse/open gender was significantly associated with depressive symptoms in the regression model. For generalized anxiety, female and diverse/open gender emerged as a risk factor in both pandemic years, while for loneliness, female and diverse/open gender seemed to play a subordinate role. Being a first-year student was significantly associated with all parameters indicating mental burden in both pandemic years, except for generalized anxiety in 2021. Having a partnership emerged as a protective factor for depressive symptoms and loneliness in 2020 and 2021. Living with others revealed negative associations with depressive symptoms in both pandemic years as well as with generalized anxiety in 2021. Pandemic-related social stress and health issues were risk factors for all mental health parameters throughout the pandemic measurements.

**Table 2:** Results of the multiple regression analyses on depressive symptoms, anxiety, and loneliness in 2020 and 2021.

	**2020**					**2021**				
	***B***	***Stand B***	**95%CI**	***t***	***p*** **-value**	***B***	***Stand B***	**95%CI**	***t***	***p*** **-value**
**Depressive symptoms**	(*R*^2^ = 0.20)					(*R*^2^ = 0.19)				
Female gender	0.59	0.05	0.15, 1.02	2.65	**0.008**	0.60	0.05	−0.12, 1.32	1.65	0.100
Diverse/open gender	3.95	0.06	1.62, 6.28	3.32	**< 0.001**	4.93	0.13	2.88, 6.99	4.71	**< 0.001**
First study year	0.84	0.06	0.38, 1.30	3.59	**< 0.001**	1.03	0.07	0.29, 1.78	2.72	**0.007**
In a partnership	−0.85	−0.08	−1.23, −0.46	−4.33	**< 0.001**	−0.63	−0.05	−1.24, −0.03	−2.06	**0.040**
Living with others	−0.72	−0.04	−1.31, −0.12	−2.36	**0.019**	−1.39	−0.08	−2.27, −0.51	−3.11	**0.002**
Pandemic-related social stress	0.19	0.12	0.13, 0.26	6.14	**< 0.001**	0.20	0.12	0.10, 0.30	3.98	**< 0.001**
Pandemic-related health issues	0.64	0.36	0.57, 0.70	19.26	**< 0.001**	0.62	0.33	0.51, 0.73	11.11	**< 0.001**
**Generalized anxiety**	(*R*^2^ = 0.17)					(*R*^2^ = 0.16)				
Female gender	0.38	0.010	0.24, 0.52	5.26	**< 0.001**	0.25	0.06	0.03, 0.47	2.22	**0.027**
Diverse/open gender	1.64	0.08	0.89, 2.40	4.26	**< 0.001**	1.20	0.10	0.57, 1.84	3.74	**< 0.001**
First study year	0.17	0.04	0.02, 0.32	2.22	**0.026**	0.12	0.03	−0.11, 0.35	1.00	0.320
In a partnership	−0.09	−0.03	−0.22, 0.03	−1.49	0.136	0.12	0.03	−0.07, 0.30	1.22	0.221
Living with others	−0.05	−0.01	−0.24, 0.15	−0.49	0.626	−0.29	−0.06	−0.56, −0.02	−2.10	**0.036**
Pandemic-related social stress	0.05	0.010	0.03, 0.07	5.09	**< 0.001**	0.04	0.07	0.01, 0.07	2.31	**0.021**
Pandemic-related health issues	0.18	0.33	0.16, 0.20	16.76	**< 0.001**	0.19	0.34	0.16, 0.23	11.13	**< 0.001**
**Loneliness**	(*R*^2^ = 0.29)					(*R*^2^ = 0.24)				
Female gender	0.04	0.01	−0.17, 0.25	0.39	0.695	0.05	0.01	−0.32, 0.41	0.26	0.795
Diverse/open gender	1.02	0.03	−0.12, 2.16	1.76	0.078	1.30	0.07	0.27, 2.34	2.46	**0.014**
First study year	0.47	0.07	0.24, 0.69	4.10	**< 0.001**	0.54	0.07	0.17, 0.92	2.83	**0.005**
In a partnership	−0.75	−0.13	−0.93, −0.56	−7.86	**< 0.001**	−0.73	−0.12	−1.04, −0.42	−4.68	**< 0.001**
Living with others	0.02	0.00	−0.27, 0.31	0.14	0.889	−0.25	−0.03	−0.69, 0.20	−1.08	0.281
Pandemic-related social stress	0.38	0.45	0.35, 0.41	24.93	**< 0.001**	0.33	0.38	0.28, 0.38	13.25	**< 0.001**
Pandemic-related health issues	0.12	0.13	0.09, 0.15	7.43	**< 0.001**	0.14	0.14	0.08, 0.19	4.88	**< 0.001**

Stand B, standardized B. CI, Confidence interval (lower CI, upper CI). Bold values indicate statistical significance.

## Discussion

The present study investigated the mental burden among students from 2019 to 2021, based on three cross-sectional surveys conducted during the respective summer semesters. We add to previous research by comparing two pandemic measurements with the pre-pandemic assessment and by including gender. The proportion of students with clinically relevant depressive symptoms was significantly higher during the pandemic (38.9% in 2020, and 40.7% in 2021), compared to pre-pandemic (29.0% in 2019). Similarly, the proportions of generalized anxiety in 2020 and 2021 were significantly higher than in pre-pandemic. In addition, the level of loneliness was significantly higher in 2020 compared to 2019 and remained at a high level in 2021. The high and increasing proportions of students reporting any level of suicidal ideation (from 16.0% in 2019 to 21.4% in 2021) raise strong concerns, implying that every fifth student is severely burdened. Our findings on pre-pandemic mental health (2019) have already pointed to high distress in the student sample. However, comparing mental health before and during the pandemic indicates that mental health was strongly adversely affected, not only in the first year of the pandemic but also over the further course. In addition to female and diverse/open gender, regression analyses emphasized that being single, living alone, being a first-year student, and pandemic-related health issues and social stress were risk factors associated with increased mental burden during the pandemic.

Regarding mental burden due to the pandemic, our findings align with previous research that revealed increases in depressive symptoms, anxiety, loneliness, and suicidal ideation [e. g., (15, 16)]. A further increase in depressive symptoms during the pandemic is in line with the results of a German survey comparing mental health in 2020 with 2021 (26). These findings suggest that the mental burden remained high in the second year of the pandemic. Most universities have returned to face-to-face teaching no sooner than in the summer semester of 2022, implying that normal university life has been interrupted for 2 years. Although both years, 2020 and 2021, have been affected by the COVID-19 pandemic, different challenges may have impacted mental health in these 2 years. While the novelty of the pandemic situation characterized 2020, which came along with uncertainties due to a lack of knowledge regarding the virus, and previously unknown mobility restrictions, 2021 was challenging as the return to “normality” took place more slowly than expected.

The implementation of protective measures, especially physical distancing, has impacted everyday life. A systematic review and meta-analysis by Ernst et al. (33) reported an increase in loneliness since the start of the pandemic. However, social connectedness is a crucial protective factor for mental health (42–44) and reduces the risk of mental distress due to social isolation among students (45). In addition to the significant adverse effects of the pandemic on social life, financial uncertainties due to job loss, disrupted academic plans such as postponed internships or studies abroad, and potentially stressful living conditions (e.g., moving back to the parents and loss of autonomy) represent further challenges for students during the pandemic (46). Another factor that specifically applies to the university context might be that students needed high levels of self-management and discipline, as many courses shifted to online classes. While in the short-term, this flexible teaching format might have been perceived as beneficial by students, it may have been increasingly exhausting to maintain high levels of the necessary self-discipline over time. Furthermore, in Germany, students were among the last to get access to vaccination due to their young age (47).

Looking at variables associated with mental burden, the female gender turned out as a risk factor for depressive symptoms and anxiety during the pandemic, which is in line with previous findings in general (4, 5, 28), and in the student population (7, 12, 48, 49). It appears surprising that the female gender was not significantly associated with depressive symptoms in the 2021 regression model, as the ANOVAs revealed significantly higher levels in female participants, compared to male participants (Figure 1). We assume that the effect of the female gender on depressive symptoms in 2021 turned out insignificant due to correlations with the first-year and partnership variable in the regression model.

In terms of diverse/open gender as a risk factor for mental health, our results revealed significantly higher values in depressive symptoms, anxiety, loneliness, and suicidal ideation in diverse/open gender participants, thus corroborating the increased vulnerability of this student subgroup reported in previous studies (8, 26). Worse mental health outcomes in marginalized populations (such as diverse/open-gender individuals) due to discrimination, rejection, and stigmatization are well-documented (50, 51), which helps understand the significant difference between diverse and female/male students in all three surveys. Future research should particularly focus on these subgroups' needs in the university context and possibilities to provide adequate psychosocial support. Moreover, the results underscore the importance of creating safe spaces for marginalized populations and promoting diversity in universities.

Being a first-year student also turned out to be associated with elevated depressive symptoms, anxiety, and loneliness, while having a partnership and living with others emerged as protective factors. Regarding elevated mental distress among first-year students, our results are in line with prior findings. Perissotto et al. (52) investigated mental health in medical students during the pandemic and reported higher levels of mental burden in first-year students, assuming that these students may have fewer resources to deal with stress. Furthermore, students socialize and find friendships and relationships at the university during curricular and extracurricular activities, e.g., in campuses, bars, and other venues that promote social networking. Thus, it is not surprising that especially those students who lacked personal experience of university life and lived alone and without a steady relationship were adversely affected. This finding underlines the relevance of social events in the university context, especially for first-year students with little study experience and the lack of a social network in the new university environment. Furthermore, it highlights the importance of not closing universities too easily in times of challenging circumstances.

Pandemic-related social stress and health issues were associated with mental burden in both pandemic measurements, with comparably high standardized coefficients underlining their predictive value. The more the students reported pandemic-related social stress and health issues, the more they reported depressive symptoms, generalized anxiety, and loneliness. While depressive symptoms and anxiety were stronger associated with health issues, loneliness was stronger associated with social stress. With pandemic-related health issues and social stress being associated with mental distress in both pandemic years, our findings again emphasize the burdensome effect of the pandemic situation and the protective measures on students' mental health.

The results of the current study need to be interpreted in light of several limitations. Since we conducted a cross-sectional analysis, we can only report the proportions of mentally distressed students across the 3 years, but no intrapersonal changes. Therefore, we cannot draw causal conclusions. Furthermore, our study sample included *n* = 95 participants who participated in all three surveys. However, we did not exclude participants with repeated measures from analyses and we treated the data from the three surveys as independent data. Another limitation is the low response rate, which decreased from 14.0% in 2019 to 4.7% in 2021. Furthermore, generalizability is limited due to several reasons. We noticed an over-representation of female participants, pointing to a common issue in health research (53). Additionally, findings on diverse/open-gender participants need to be interpreted with caution, as this subsample is comparably small. Furthermore, we summarized responses of the “diverse” and “open” responses in 2021 into one category due to low sample sizes, which may weaken the validity of the findings. In terms of selection bias, we assume that especially students who were interested in health issues might have subscribed to study participation. Finally, seasonal variations in COVID-19-related restrictions and protective measures weaken the generalizability of the current results to other countries, as we analyzed data collected at a single university in Germany. However, with its large database across 3 years, the current study provides valuable insights into mental health among students at the JGU Mainz during the pandemic. By reporting two pandemic measure times, both collected in summer months, our study design allows comparing mental adjustment to the pandemic among university students in two subsequent years. Including participants with diverse/open gender in statistical inference analysis is often neglected in research but revealed important findings in our study sample.

### Practical implications

Even though the pandemic is currently on the decline and universities have mostly resumed regular operations (i.e., face-to-face classes) since the summer semester of 2022, the impact of the pandemic on student mental health should still be considered. Teaching personnel should be made aware of the increased mental distress of students during the pandemic and be able to refer them to professional support services as needed. Psychological symptoms should also be discussed with the students themselves. Using psychoeducational methods, students can be supported to become aware of their own symptoms and to check whether, for example, a prevention course is sufficient for their needs or whether they need more intensive support (e.g., psychological support and psychotherapeutic treatment). Low-threshold psychological counseling services at the university should be further expanded and promoted. With respect to diverse/open-gender students, universities should consider the extent to which study conditions and support services are free of discrimination. In addition, specific offerings for diverse/open-gender students may be a way to pay more attention to this subgroup and its needs.

## Conclusion

In a growing database, our findings show that the closure of universities meant for the protection of health came at high long-term mental health costs for students, particularly for vulnerable subpopulations. The results of the current study imply that the mental burden among students continued through the second year of the pandemic, with higher proportions of students with clinically relevant depressive symptoms and anxiety and students reporting any level of suicidal ideation. Gender-sensitive analyses revealed increased mental burden, especially in female and diverse/open-gender students. Furthermore, first-year students, singles, and students who live alone were particularly vulnerable to mental burdens during the pandemic. It is unknown how well students have adjusted regarding general health, academic achievement, career, financial and living conditions, since reopening the universities. Future research should monitor recovery and evaluate needs for psychosocial support.

## Data availability statement

The raw data supporting the conclusions of this article will be made available by the authors, without undue reservation.

## Ethics statement

The studies involving human participants were reviewed and approved by the Ethical Committee of the Medical Association of Rhineland-Palatinate (No. 2019-14336) and the Ethical Committee of the Institute of Psychology of the Johannes Gutenberg-University Mainz (No. 2020-JGU-psychEK-S008 and 2021-JGU-psychEK-S017). Written informed consent from the participants' legal guardian/next of kin was not required to participate in this study in accordance with the national legislation and the institutional requirements.

## Author contributions

ATs, AW, ATi, and MB analyzed the data. ATs, AW, and MB were the major contributors in the writing process. AW, ATi, LM, JR, SH, MS, LS, TR, BS, PD, and MB were involved in the development and performance of the survey. All authors contributed in writing the manuscript, read, and approved the final manuscript.
